# Quality of life in patients with advanced renal cell carcinoma treated with temsirolimus or interferon-*α*

**DOI:** 10.1038/sj.bjc.6605647

**Published:** 2010-05-11

**Authors:** S Yang, P de Souza, E Alemao, J Purvis

**Affiliations:** 1 Global Access, Pfizer, 500 Arcola Road, Collegeville, Pennsylvania, USA; 2 Cancer Care Centre, St George Hospital Clinical School, Gray Street, UNSW, Kogarah, New South Wales, Australia; 3 Global Clinical Development, (Oncology), Otsuka Pharmaceutical Development & Commercialization, Inc., Princeton, NJ 08540, USA

**Keywords:** interferon, quality of life, renal cell carcinoma, temsirolimus

## Abstract

**Background:**

Temsirolimus was approved in Europe as first-line treatment of poor-prognosis advanced renal cell carcinoma (advRCC) based on significant clinical benefits.

**Methods:**

Patients with advRCC and multiple poor-prognostic factors were randomly assigned to receive 25 mg intravenous temsirolimus weekly, interferon-*α* (titrated to 18 mU) three times weekly, or 15 mg intravenous temsirolimus weekly plus 6 mU of interferon-*α* three times weekly. EuroQol-5D utility score (EQ-5D index) and the EQ-5D visual analogue scale (EQ-VAS) responses were recorded. For analysis, patients were required to have their EQ-5D data recorded at baseline, week 12, and last visit after week 12. The analysis was conducted using last-visit data and a repeated-measures mixed-effect (RMME) model to evaluate quality-of-life differences between temsirolimus and interferon-*α*, controlling for baseline covariates.

**Results:**

Average EQ-5D score at the last measure was significantly higher in patients receiving temsirolimus compared with interferon-*α*: by 0.10 on EQ-5D index (*P*=0.0279) and by 6.61 on EQ-VAS (*P*=0.0095). In the RMME model, the least-square mean for on-treatment EQ-5D index score was 0.590 with temsirolimus and 0.492 with interferon-*α* (*P*=0.0022).

**Conclusion:**

Temsirolimus is associated with significantly higher EQ-5D scores compared with interferon-*α* in patients with previously untreated poor-prognosis advRCC.

Renal cell carcinoma (RCC) is the most common and lethal form of kidney cancer, accounting for 80–90% of renal cancers and 2.6% of adult cancers (Mathew *et al*, 2002; Hudes *et al*, 2007; Wein *et al*, 2007; Cella *et al*, 2008; Jemal *et al*, 2008). Renal cell carcinoma is twice as frequent in men as in women, and most commonly affects patients aged 50–80 years. More than 40% of patients with RCC die of the cancer, compared with 20% of patients with prostate or bladder cancer (Wein *et al*, 2007). Only 20% of patients with advanced RCC (advRCC) survive for 5 years (Cella *et al*, 2008).

Advanced RCC is refractory to conventional chemotherapy (Brugarolas, 2007; Motzer *et al*, 2007) and has long been managed with cytokine-based therapies (van Spronsen *et al*, 2005). Interferon-*α* (IFN-*α*) prolongs overall survival in some patients (van Spronsen *et al*, 2005). Adverse events with IFN-*α* can impact quality of life (QOL).

The advent of targeted therapies has contributed to a paradigm shift in metastatic RCC management (Coppin *et al*, 2008). Temsirolimus (CCI-779 or Torisel, Pfizer, New York, NY, USA) is a specific inhibitor of the mammalian target of rapamycin kinase that provides survival benefits as first-line therapy in patients with advRCC and a poor prognosis (Brugarolas, 2007; Hudes *et al*, 2007; Coppin *et al*, 2008; Motzer *et al*, 2008). By inhibiting mammalian target of rapamycin kinase, temsirolimus reduces translation of a variety of messenger RNA, including those for key transcription factors, which is a step in the signalling cascade for angiogenesis and has been implicated in the development of clear-cell renal tumours.

In the international, randomised, phase III study (Global Advanced Renal Cell Carcinoma (ARCC) trial; ClinicalTrials.gov study no. NCT00065468; Hudes *et al*, 2007), temsirolimus was associated with a significant overall survival benefit compared with IFN-*α* (median overall survival=10.9 *vs* 7.3 months; hazard ratio for death=0.73 (95% confidence interval (CI)=0.58, 0.92); *P*=0.008) and progression-free survival benefit as first-line treatment for poor-prognosis advRCC. Quality-adjusted survival data, assessed by quality-adjusted time without symptoms of progression or toxicity (Q-TWiST), have been reported previously (Zbrozek *et al*, 2010). The objective of this study was to evaluate, among patients in the Global ARCC trial, QOL as assessed by the EuroQol-5D utility score (EQ-5D index) and the EQ-5D visual analogue scale (EQ-VAS), which were two protocol-specified questionnaires administered during the trial.

## Patients and methods

### Treatments

This study is an analysis reporting QOL data among a subgroup of 626 patients with previously untreated, poor-prognosis advRCC from Global ARCC (Hudes *et al*, 2007; National Institutes of Health, 2007). Eligible patients were enroled between July, 2003 and April, 2005, and randomly assigned to 25 mg of intravenous (i.v.) temsirolimus weekly or 3–18 mU of subcutaneous IFN-*α* (IFN-*α*2a; Roferon-A, Roche Pharmaceuticals, Nutley, NJ, USA) three times weekly (Roferon-A, 2008 (US full prescribing information)). A third group was randomised to combination therapy, but was not included in this analysis because there was no survival advantage.

### Patients

Eligible patients had histologically confirmed advRCC (stage IV or recurrent disease); a Karnofsky performance score of ⩾60; no previous systemic treatment; a measurable tumour according to response evaluation criteria in solid tumours (RECIST; Therasse *et al*, 2000); adequate bone marrow, kidney, and liver functions; and fasting levels of total cholesterol and triglyceride below specified levels (Hudes *et al*, 2007). Patients were required to have at least three of six predictors of short survival to qualify as having poor prognosis. The six predictors included the five Memorial Sloan-Kettering Cancer Center (MSKCC) prognostic factors: <1 year from time of initial RCC diagnosis to randomisation; a Karnofsky performance status of 60 or 70; haemoglobin level less than the lower limit of normal; corrected calcium >2.5 mmol l^−1^; and serum lactate dehydrogenase >1.5 times the upper limit of normal (Motzer *et al*, 1999, 2002; Mekhail *et al*, 2005). The remaining predictor was more than one metastatic organ site of disease (Mekhail *et al*, 2005).

### QOL assessments

Self-reported QOL was evaluated using the EQ-5D questionnaire (The EuroQol Group, 1990; Oppe *et al*, 2008). It is designed to cover common areas of health QOL (Brooks, 1996). The EQ-5D was found to have responsiveness comparable to that of the European Organisation for Research and Treatment of Cancer Quality of Life Questionnaire C-30 (Krabbe *et al*, 2004). The EQ-5D is particularly well suited for international trials because it has been validated and translated into more than 100 languages (Oppe *et al*, 2008). It consists of two pages: the first with descriptive questions that generate the EQ-5D index score (the utility score) and the second with the EQ-VAS (Oppe *et al*, 2008).

The EQ-5D descriptive system has five dimensions: mobility, self-care, usual activities, pain/discomfort, and anxiety/depression (Oppe *et al*, 2008). Patients can respond to a question on each dimension by marking a box corresponding to ‘no problems,’ ‘some problems,’ or ‘severe problems’ (scored as 1, 2, and 3, respectively). These numerals do not have arithmetic properties and cannot be used as a cardinal scale. A five-digit number can be created by combining the answers to the five dimensions, and thus 243 health states can be described using this system (Oppe *et al*, 2008). The EQ-VAS allows patients to rate their health by drawing a line on a thermometer-like scale from 0–100, in which 0 is the worst possible health status and 100 the best health condition (Greiner *et al*, 2003; Oppe *et al*, 2008). It has acceptable validity and excellent reliability as a global QOL measure for clinical trials (de Boer *et al*, 2004). The EQ-5D was scored using the index-based algorithm as described by Dolan (1997).

In this study, patient QOL was measured using the EQ-5D index and EQ-VAS at screening, week 12, week 32, any visit at which the patient reported a symptomatic National Cancer Institute Common Terminology Criteria, version 3.0 grade 3 (severe) or 4 (life-threatening or disabling) adverse event (unless the medical condition prohibited using the EQ-5D; National Cancer Institute, 2006), and the withdrawal visit. In this analysis, EQ-5D measures at week 12 and the last measure were used wherever possible. For patients with a withdrawal visit recorded, the last measure was defined as the EQ-5D at the withdrawal visit. For those without a withdrawal visit recorded, the visit at which the last EQ-5D was recorded was the last measure. Patients with EQ-5D at screening only or with EQ-5D data up to week 12 only were excluded from the analyses.

### Statistical analysis

Two types of statistical analyses were performed. An analysis of outcomes between the two treatments at last study visit was conducted, analysing the overall population and subgroups on the basis of prior nephrectomy status, primary cell type, and a number of poor-prognosis factors at baseline. For each subgroup, mean EQ-5D index score and mean EQ-VAS score at last visit by treatment were computed, as were their differences along with the 95% CIs of the differences. Two-tailed *t*-tests at *α*=0.05 were conducted for independent samples to compare outcomes. The null hypothesis was that there were no differences between the two treatment groups.

The second analysis evaluated mean EQ-5D VAS and index measures between two treatments using a model controlling for age, gender, baseline QOL, tumour histology type, MSKCC prognostic factor status, prior nephrectomy history, and time since randomisation. Repeated measures indicate multiple responses taken at different times from the same patients (Littell *et al*, 1998). Data analysis of repeated measures permits a comparison of response trends over time by treatment. The mixed-model methodology is particularly appropriate because it accounts for covariance issues in multiple measures of patients’ responses over time. Other advantages of repeated-measures mixed-effect (RMME) models include their superior flexibility in modelling time effects, ability to account for correlations among repeated assessments of the same person, and capacity to account for missing data (Gueorguieva and Krystal, 2004).

## Results

### Baseline characteristics

Patients were randomly assigned to temsirolimus (*n*=209) or IFN-*α* (*n*=207) groups. Of these 416 patients, 270 (65%) were evaluable for QOL analysis: 155 for temsirolimus and 115 for IFN-*α*. The two treatment groups were well balanced on the basis of age, sex, and other characteristics at baseline (Supplementary Online Table 1), and no baseline characteristic was significantly different between the two groups (Hudes *et al*, 2007). The average patient age was 59 years (s.d.=10). Approximately 32% of the patients were females, 85% had clear-cell carcinoma, and 95% had at least three poor-prognostic factors. The mean EQ-5D utility score (s.d.) was 0.62 (0.24), and the mean EQ-5D VAS score (s.d.) was 64.03 (17.17). Approximately 80% of patients in each group had a Karnofsky performance score of 60 or 70; received a diagnosis of metastatic RCC within 12 months of enrollment; haemoglobin below lower limit of normal; and two or more sites of organ metastases. Approximately two-thirds of the patients in each treatment group had undergone nephrectomy.

### Last-visit analysis

**Figure 1:**
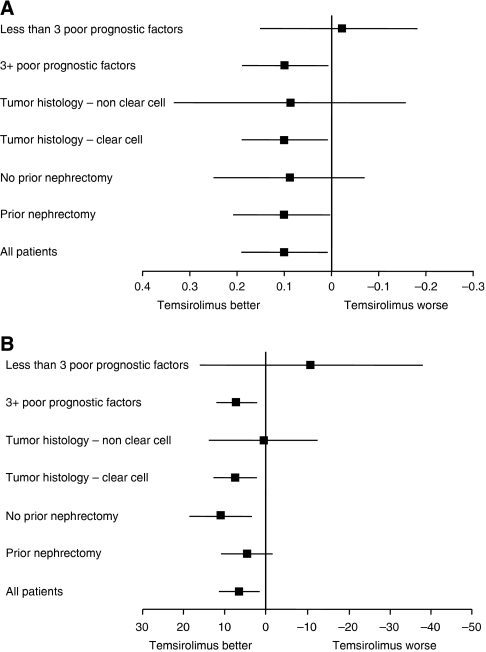
(**A**) Temsirolimus *vs* IFN-*α* mean (95% CI) differences in EQ-5D index scores at the last study visit. (**B**) Temsirolimus *vs* IFN-*α* mean (95% CI) differences in EQ-VAS scores at the last study visit. For tumour histology, the ‘non clear cell’ category includes some patients with indeterminate histology. Solid horizontal lines, 95% CIs; solid vertical lines, no difference. CI=confidence interval, EQ-5D=EuroQol Group's 5-dimension questionnaire; EQ-VAS=EQ-5D visual analogue scale; IFN=interferon.

In the last-visit analysis of EQ-5D index scores, temsirolimus was associated with superior EQ-5D index scores compared with IFN-*α* (Figure 1A). The mean EQ-5D index score at last measure was higher in the temsirolimus arm than in the IFN-*α* arm by 0.10 (*P*=0.0279). Similarly, patients with clear-cell tumour histology treated with temsirolimus had mean index scores significantly higher than those treated with IFN-*α* (*P*=0.0395). Patients with at least three poor-prognostic factors treated with temsirolimus also had mean index scores significantly higher than those treated with IFN-*α* (*P*=0.0308).

In a corresponding (last-visit) analysis of EQ-VAS scores, temsirolimus was associated with superior EQ-VAS scores compared with IFN-*α* (Figure 1B). The mean EQ-VAS score at last measure was higher in the temsirolimus arm than the IFN-*α* arm by 6.61 (*P*=0.0095). In the last-visit analysis of mean EQ-VAS scores, scores were significantly higher in the temsirolimus arm compared with the IFN-*α* arm among patients with no prior nephrectomy (*P*=0.0056) or with clear-cell histology (*P*=0.0061), and/or at least three poor-prognostic factors (*P*=0.0056).

### RMME analysis

In the RMME model, temsirolimus was also associated with superior EQ-5D index scores compared with IFN-*α*. The least-square mean for on-treatment EQ-5D index scores in the IFN-*α* arm was 0.492 (s.e.=0.031) and in the temsirolimus arm was 0.590 (s.e.=0.026; *P* value for difference=0.0022 (95% CI=–0.162, –0.036)). The other significant covariates (Table 1) were as follows: patients in the MSKCC intermediate-risk group had on-treatment EQ-5D index scores higher than those in the poor-risk group (*P*<0.0001 (0.080, 0.214)); patients assessed at week 12 had higher on-treatment EQ-5D index scores compared with patients at the withdrawal visit (*P*<0.0001 (0.055, 0.148)); and patients having higher baseline EQ-5D index scores also had higher on-treatment EQ-5D scores (*P*<0.0001 (0.470, 0.733)).

Temsirolimus was associated with significantly greater EQ-5D VAS scores compared with IFN-*α*: the least-square mean for the on-treatment EQ-5D VAS score in the IFN-*α* arm was 58.83 (s.e.=1.83) and in the temsirolimus arm was 63.33 (s.e. =1.56; *P*=0.0168; 95% CI=−8.184, −0.819). As summarised in Table 1, other significant covariates predicting higher on-treatment EQ-5D VAS scores included high EQ-5D VAS at baseline, patient placement in the MSKCC intermediate-risk group, with no prior nephrectomy, and patient assessment at week 12 compared with those assessed at their withdrawal visit.

## Discussion

This study, to the best of our knowledge, is the first to compare EQ-5D scores for temsirolimus and IFN-*α* in previously untreated patients with advRCC and multiple poor-prognostic factors. Temsirolimus was associated with significantly higher (improved) patient-reported EQ-5D compared with IFN-*α*. At the last study visit, patients treated with temsirolimus had higher mean EQ-5D index and EQ-VAS scores compared with those treated with IFN-*α*. Consistent with these results, patients treated with temsirolimus (*vs* IFN-*α*) also had higher mean EQ-5D index and EQ-VAS scores in an RMME analysis. The difference in EQ-5D index scores between temsirolimus and IFN-*α* was 0.099. This was greater than the minimally important difference. The minimally important difference was first established at 0.05 as a general rule, based on 5% of the instrument's maximal score, or as 0.06–0.08 depending on the country (Pickard *et al*, 2007; Ringash *et al*, 2007). Thus, these results for temsirolimus should be considered clinically and statistically significant.

These findings complement data from the parent Global ARCC study that found a significant prolongation of overall survival with temsirolimus compared with IFN-*α* (Hudes *et al*, 2007). Our analysis also extends to findings from the Q-TWiST analysis (Zbrozek *et al*, 2010), which demonstrated that patients with advanced, metastatic RCC receiving temsirolimus have significantly longer quality-adjusted survival (by 1.4 months; ∼25% increase in Q-TWiST) compared with patients treated with IFN-*α*. As Q-TWiST is an index of time spent in different health states and is not treatment specific, Q-TWiST analyses can result in the same or a very similar score for a patient with a grade 3 or 4 adverse event irrespective of whether the event occurred during temsirolimus or IFN-*α* therapy. As the present analysis is treatment specific, it can serve as a foundation for establishing treatment-specific utilities for cost-effectiveness analyses. Our examination of the differences in EQ-5D scores in the two treatment arms suggests that the increase in quality-adjusted survival associated with temsirolimus was not a mere artefact of an increased survival time but was instead a function of the therapy itself.

The RMME models controlling for covariates determined that the only factors associated with higher on-treatment EQ-5D index scores were temsirolimus, baseline EQ-5D index scores, MSKCC intermediate-risk status, and assessment at treatment week 12. (Besides the above factors, patients with no prior nephrectomy also had higher EQ-5D VAS scores.) To the best of our knowledge, this study is the first to report that MSKCC intermediate-risk status (presence of one or two of the five MSKCC risk factors) was associated with significantly higher EQ-5D scores than poor-risk status (presence of at least three MSKCC risk factors; Motzer *et al*, 1999). This finding strengthens the rationale for using the MSKCC risk category as a stratification variable or a covariate in clinical trial designs. Memorial Sloan-Kettering Cancer Center risk factors are used to stratify patients into three different groups: favourable risk, intermediate risk, and poor risk (Motzer *et al*, 1999). The 3-year survival rates for these groups in the MSKCC study were 31%, 7%, and 0%, respectively (Motzer *et al*, 1999). However, because this study had only 14 patients (five in the IFN-*α* arm and nine in the temsirolimus arm) with less than three risk factors, this finding may not be generalisable to a larger population that includes patients with one or two risk factors.

The instruments chosen to document QOL are not cancer specific because the study was designed to provide data for cross-disease economic analyses. The EQ-5D allows the patient to rate 243 different health states on a scale from 1 (no health problems) to 3 (extreme health problems), and the scores can be used to determine ‘time trade-offs’ between disease states that facilitate cost-effectiveness and cost-utility analyses. To our knowledge, the only head-to-head comparison of treatment for metastatic RCC with a targeted therapy (sunitinib) compared with IFN-*α* used the EQ-5D index and EQ-VAS scores, and the data were consistent with other findings using disease-specific instruments, including the Functional Assessment of Cancer Therapy-General, and the Functional Assessment of Cancer Therapy-Kidney Symptom Index-15 item (Cella *et al*, 2008).

There was an imbalance in the number (percent) of patients in the Global ARCC trial who survived, had evaluable EQ-5D data, and qualified for study enrollment: 155 (74.2%) of 209 original patients in the temsirolimus arm compared with 115 (55.6%) of 207 in the IFN-*α* arm. However, the RMME model was designed, in part, to limit the bias associated with uneven patient attrition in the two groups; hence, it is reassuring that findings from both analyses were consistent. Furthermore, an analysis of the causes of treatment withdrawals (data not shown) found that most (∼85%) of the last visits were occasioned by disease progression and/or symptomatic deterioration rather than adverse events (∼10%). Even if all 10% of the adverse event-related treatment withdrawals had been observed in the IFN-*α* arm, thereby reducing the QOL associated with this treatment, it is unlikely that this small proportion would affect the overall conclusions. It is also unlikely, therefore, that this factor in particular, or selective attrition in general, would be a substantial source of bias.

Our study evaluated patients at the latest possible point (last visit) rather than at a fixed time from inception of treatment. Thus, it is possible that patient-reported QOL is lower in this study than in studies that used a fixed time for evaluation, rendering cross-study comparisons more hazardous than usual. Finally, temsirolimus has not been compared head-to-head with other targeted treatments in active-comparator trials.

## Conclusion

Temsirolimus was associated with significantly higher (superior) EQ-5D scores compared with IFN-*α* in previously untreated patients with poor-prognosis advRCC. The improvement in QOL compared with IFN-*α*, along with the previously established overall survival and progression-free survival benefits of temsirolimus (*vs* IFN-*α*), suggests that the inhibitor of mammalian target of rapamycin kinase could be used as first-line therapy in such patients.

## Figures and Tables

**Table 1:** RMME of factors affecting quality of life

**RMME results for EQ-5D index scores**			
**Factors**	**Coefficient** a	***P* value**	**95% CI**
Significant impact*			
Baseline EQ-5D index score	0.601	<0.0001	(0.470, 0.733)
Treatment, IFN-*α* (*vs* temsirolimus)	−0.099	0.0022	(−0.162, −0.036)
Intermediate (*vs* poor) MSKCC risk	0.147	<0.0001	(0.080, 0.214)
Time of assessment, week 12 (*vs* withdrawal) visit	0.102	<0.0001	(0.055, 0.148)
			
*No significant impact*			
Age	–0.002	0.1495	(−0.005, 0.0008)
Female (*vs* male) gender	–0.046	0.1731	(−0.111, 0.020)
No prior (*vs* prior) nephrectomy	0.050	0.1383	(−0.016, 0.116)
Clear-cell (*vs* non-clear-cell) tumour histology type	0.011	0.7940	(−0.074, 0.097)
			
**RMME results for EQ-5D VAS scores**			
**Factors**	**Coefficient** a	**95% CI**	
Significant impact*			
Baseline EQ-5D VAS score	0.546	(0.439, 0.653)	
Intermediate (*vs* poor) MSKCC risk	6.365	(2.381, 10.349)	
No prior (*vs* prior) nephrectomy	7.220	(3.349, 11.090)	
Treatment, IFN-*α* (*vs* temsirolimus)	−4.501	(−8.184, −0.819)	
Time of assessment, week 12 (*vs* withdrawal) visit	6.080	(3.372, 8.787)	
		
*No significant impact*			
Age	−0.126	(−0.307, 0.054)	
Female (*vs* male) gender	0.150	(−3.700, 4.000)	
Clear-cell (*vs* non-clear-cell) tumour histology type	0.968	(−4.113, 6.049)	

Abbreviations: CI=confidence interval; EQ-5D=EuroQol Group's 5-dimension questionnaire; IFN=interferon; MSKCC=Memorial Sloan-Kettering Cancer Center; RMME=Repeated-measures mixed-effect model; VAS=visual analogue scale.

a For each continuous variable, the coefficient represents the quality of life per unit change in the variable. For example, after controlling for baseline covariates, an increase in age of 1 year reduced the quality of life by 0.002. For each categorical variable, the coefficient represents the quality of life as compared with the reference group, which is enclosed in parentheses. For example, patients treated with IFN-*α* had a lower quality of life by 0.099 compared with those treated with temsirolimus (reference group) after controlling for baseline covariates.

*P<0.05.
