# Supplementary material for: Quality of life in patients with advanced renal cell carcinoma treated with temsirolimus or interferon-α
Source: Br J Cancer. 2010 May 11;102(10):1456–60. doi: 10.1038/sj.bjc.6605647 (PMC2869160; doi:10.1038/sj.bjc.6605647)
Supplement: Supplementary Online Table [file 6605647x1.doc]

**Supplemental Online Table 1.**

**Baseline characteristics (by treatment groups) of patients included in the current analysis**

| **Characteristic** |  | **IFN-alfa  (n = 115)** | **TEMSR  (n = 155)** | ***P* value** | **Total = 270** |
| --- | --- | --- | --- | --- | --- |
| Age | Mean (SD) | 60 (10) | 58 (10) | 0.0585 | 59 (10) |
| Gender | Female – no. (%) | 37 (32%) | 49 (32%) | 0.9221 | 86 (32%) |
| Tumor histologic type no. (%) | Clear-cell – no. (%) | 99 (86%) | 130 (84%) | 0.6159 | 229 (85%) |
| ≥ 3 poor prognostic factors | Yes – no. (%) | 110 (96%) | 146 (94%) | 0.5930 | 256 (95%) |
| Quality of life | Mean EQ-5D utility score (SD) | 0.63 (0.23) | 0.62 (0.24) | 0.8527 | 0.62 (0.24) |
|  | Mean EQ-5D VAS  Score (SD) | 62.80 (17.76) | 64.95 (16.71) | 0.3103 | 64.03 (17.17) |
| Karnofsky score | 60-70 – no. (%) | 97 (84%) | 124 (80%) | 0.3594 | 221 (82%) |
| Hgb | Hgb < LLN – no. (%) | 92 (80%) | 126 (81%) | 0.7903 | 218 (81%) |
| Organ metastases | ≥2 – no. (%) | 93 (81%) | 125 (81%) | 0.6726 | 218 (81%) |
| Prior nephrectomy status | Yes – no. (%) | 76 (66%) | 107 (69%) | 0.6086 | 183 (68%) |
| Time from initial diagnosis to randomisation | <12 mo. – no. (%) | 88 (77%) | 126 (81%) | 0.3393 | 214 (79%) |
| Time from diagnosis to metastasis | <12 mo. – no. (%) | 93 (82%) | 135 (88%) | 0.1277 | 228 (85%) |

EQ-5D = EuroQol Group’s 5-dimension questionnaire; Hgb = haemoglobin; IFN = interferon; LLN = lower limit of normal; SD = standard deviation; TEMSR = temsirolimus; VAS = visual analog scale.
